# Sustainable Sugarcane Bagasse-Derived Activated Carbon for High-Performance Symmetric Supercapacitor Devices Applications

**DOI:** 10.3390/nano15131028

**Published:** 2025-07-02

**Authors:** Perumal Rajivgandhi, Vediyappan Thirumal, Alagan Sekar, Jinho Kim

**Affiliations:** 1 Department of Chemistry, Nehru Memorial College, Bharathidasan University, Puthanampatti, Trichy 621 007, Tamilnadu, India; rajlibniz@gmail.com; 2 Department of Mechanical Engineering, Yeungnam University, Gyeongsan-si 38541, Gyeongbuk-do, Republic of Korea; thirumalvisnu@gmail.com

**Keywords:** energy storage, sugarcane bagasse (SCB), prolysis synthesis, electrochemical measurements, supercapacitors

## Abstract

In this study, sugarcane bagasse (SCB), an abundant agricultural byproduct, was transformed into activated carbon via a controlled thermochemical pyrolysis route for high-performance energy storage applications. Herein, we utilized the activated carbon derived from pure sugarcane bagasse (SCB-AC) and further activated using KOH (SCB-KOH-AC) as an electrode material in aqueous symmetric supercapacitor configurations. The synthesized activated carbon was subjected to analysis using a range of characteristics including FT-Raman spectroscopy, which was employed to confirm the functional groups present in the carbon materials. The XPS analysis provided insights into the elemental composition and ionic states. The SEM analysis revealed that both activated carbon and KOH/activated carbon materials exhibited a layered or stacked, albeit slightly random, orientation. Electrochemical studies demonstrated that the synthesized carbon electrodes exhibited impressive specific capacitance values of (SCB) activated carbon (132.20 F/g) and KOH-activated, pure SCB AC (SCB-A) 253.41 F/g at 0.5 A/g. Furthermore, the SCB KOH-activated carbon (AC) electrode revealed a higher specific capacitance value and A//SCB-A symmetric devices delivered energy density reaching 17.91 Wh/kg and power density up to 2990 W/kg. The KOH-activated carbon electrode demonstrated remarkable cycling stability retaining 93.89%, even after 10,000 cycles. These results suggest that the sugarcane bagasse-derived activated carbon is a sustainable and low-cost candidate for next-generation supercapacitor electrodes. The results demonstrate enhanced capacitance, stability, and pore structure tailored for energy storage applications. The KOH-activated carbon SCB carbon symmetric device with symmetric electrodes exhibited a suitable bio-mass carbon for future energy storage applications.

## 1. Introduction

Activated carbon (AC) is a knowingly larger porous material with an extensive internal surface area, making it an ideal candidate for supercapacitor applications. The formation of these tiny pores is achieved by activating a carbon precursor through methods such as KOH (potassium hydroxide) activation. During this process, KOH reacts with the precursor at elevated temperatures, creating intricate pore networks and significantly enhancing the surface area [1]. Compared to standard activated carbon, KOH-activated AC features a higher surface area, adjustable pore size distribution, and potentially improved electrical conductivity due to the incorporation of potassium [2]. This increased porosity contributes to superior performance in supercapacitors. Supercapacitors serve as a bridge between batteries and traditional capacitors, offering faster charging and discharging capabilities than batteries and higher energy density than standard capacitors. Activated carbon typically decomposes into gaseous products such as carbon dioxide (CO_2_), carbon monoxide (CO), and small amounts of hydrocarbons when exposed to temperatures above 500 °C. The specific decomposition products and their proportions can vary depending on the type of activated carbon and the conditions of decomposition, such as the presence of oxygen or other reactive gases. The high surface area of AC facilitates efficient charge storage through electrostatic adsorption, where electrolyte ions accumulate within the pores, contributing to the overall capacitance of the supercapacitor. KOH activation further enhances capacitance by introducing oxygen-containing functional groups on the AC surface, which promote even better ion adsorption.

The activation of carbon using KOH has emerged as a highly effective method for enhancing the material’s properties, particularly for applications in CO_2_ capture and supercapacitors [3]. The quality of activated carbon can be significantly influenced by various operational parameters, including particle size, temperature, residence time, and heating rate [4]. Among these factors, temperature is the most critical and effective. The literature reports the yield of SCB (sugarcane bagasse)-activated carbon ranging between 14% and 56%. SCB-activated carbon has diverse applications, such as soil amendment, wastewater treatment, adsorbent in water, and accompanying cementitious material [5]. The study also highlights several gaps in knowledge, including cost wise analysis and the evaluation of using bagasse as fuel in sugar manufacturing versus producing activated carbon. Sugarcane bagasse-activated carbon has significant prospective to enhance a highly attractive carbonaceous material with wide-ranging applications across various industries.

Activated carbon is a highly potential material valued for its large surface area, high porosity, and tunable surface chemistry, making it suitable for diverse applications such as energy storage, environmental remediation, and chemical processing [6]. However, its performance characteristics depend strongly on the source material and activation method. In recent years, biomass-derived activated carbons have gained significant attention as sustainable and cost-effective alternatives to conventional carbon materials. Among these, sugarcane bagasse, a lignocellulosic agro-waste generated in large quantities by the sugar industry, has emerged as a promising precursor due to its high carbon content and renewable nature [7]. In this study, we report the synthesis of activated carbon from sugarcane bagasse using KOH chemical activation and investigate its electrochemical performance in symmetric supercapacitor devices. Unlike traditional activated carbon types, which are often optimized for filtration or adsorption (e.g., granular, powdered, and extruded forms) [8], our approach is focused on engineering the pore structure and surface properties of sugarcane-derived carbon specifically for energy storage applications. This work demonstrates the potential of converting agricultural waste into high-performance electrode materials, contributing to the advancement of environmentally friendly technologies. It is used in processes such as removing volatile organic compounds (VOCs) from air. Finally, by selecting the appropriate type of activated carbon, industries can achieve efficient and targeted removal of contaminants in various applications, including air and water purification, odor control, and even gold recovery in mining [8].

From earlier reports, sugarcane bagasse is a leftover from the sugar production process, which occurs after the sugar juice is extracted. According to the latest data, India produces 350 million metric tons of sugarcane, yielding 66.9 metric tons per hectare. For every ton of sugarcane processed, 180–280 kg of bagasse waste is generated [9]. Through environmental resource reconditioning, we employ both physical and chemical activation methods to transform this material into activated carbon with a specific structure. This activated carbon is further enhanced by combining it with potassium hydroxide (KOH) and potassium nitrate (KNO_3_), resulting in a significant increase in its specific surface area and exceptional specific capacitance (SCP). The combined electrode created from this modified carbon exhibits excellent electrical conductivity of 57 mS/cm. A supercapacitor is then constructed using a sandwich structure consisting of the electrode, gel electrolyte, and separator. At a scan rate of 0.02 V/s, the supercapacitor attains an SCP of 262.4 F/g, an energy density of 17.9 W/kg, and a power density of 2021 W/kg. Additionally, the supercapacitor exhibits remarkable cycling stability, retaining 98.38% of its original capacitance after 10,000 charge and discharge cycles at a standard current. The use of sugarcane bagasse as a carbon precursor offers significant environmental and economic advantages compared to traditional carbon sources [10]. As an agricultural waste product, SCB helps reduce waste and promotes sustainable practices in the agricultural sector. Conducting a life-cycle analysis would provide insights into the environmental benefits, including reductions in greenhouse gas emissions and resource consumption associated with SCB processing compared to other carbon precursors. Additionally, a cost–benefit analysis could demonstrate the economic viability of SCB-derived carbon, highlighting lower raw material costs and potential revenue from waste management solutions. By incorporating these analyses, the argument for the adoption of SCB as a sustainable carbon source in energy storage applications is strengthened.

The novelty of the present work lies in the comprehensive approach of utilizing sugarcane bagasse (SCB) to synthesize activated carbon (AC) and KOH-activated carbon for supercapacitor applications, coupled with extensive characterization and electrochemical performance analysis. The current investigations introduce significant advancements by employing pyrolysis and KOH activation, resulting in higher specific surface areas, enhanced electrical conductivity, and substantial improvement in specific capacitance compared to other agricultural-waste-derived carbons. Notably, the KOH activation electrode achieved remarkable specific capacitance values (499.7 F/g at 10 mV/s and 603.12 F/g at 1 A/g) and excellent cycling stability (94.7% retention after 10,000 cycles), surpassing previously reported carbon-based supercapacitors. Furthermore, the Kubelka–Munk band gap analysis (3.7 eV for AC and 2.7 eV for KOH-AC) adds a new dimension in understanding the electronic properties of these materials. This work showcases the potential of SCB as a sustainable, low-cost precursor for high-performance energy storage devices, contributing to environmental resource reconditioning and offering a novel perspective on agricultural waste utilization in advanced energy applications.

## 2. Experimental Method

Sugarcane bagasse was obtained from local sugar refineries, cut into tiny fragments, and carefully washed with deionized water. The processed bagasse was then heated in a hot air furnace at 110 °C for a duration of 12 h. After that, to manufacture KOH-activated carbon, the bagasse was subjected to carbonization after a preliminary treatment with a chemical substance using KOH. In this step, 30 g of unprocessed selective material was combined with 150 g of an 80% KOH aqueous solution by weight and allowed to marinate for 24 h. Following the marination, the samples that had undergone preliminary treatment were dried out in a hot air furnace at 110 °C for another 12 h. Both the untreated and KOH impregnated materials were then carbonized at 670 °C for 2 h in a muffle furnace, which heated at a level of 10 °C per minute. The resulting materials were rinsed with deionized water until they reached a neutral pH level and were subsequently dried at 60 °C to produce the final product.

### Electrode Preparation and Characterization

The electrode fabrications through active materials were mixed together with 80 wt% powdered materials (sugarcane bagasse-derived carbon) with 10 wt% polyvinylidene fluoride (PVDF) and 10 wt% acetylene black, utilizing a homogenous composite suitable for electrode fabrication. The active material was thoroughly mixed with the Supporting Materials (Figures S1 and S2) using an agate mortar and pestle to ensure uniform grinding and homogeneous dispersion to ensure a uniform, deep fine slurry. This slurry ink was subsequently drop-cast onto nickel foam, after which the coated electrode material was placed in an oven set at 90 °C overnight. This step was crucial for solvent removal, thereby ensuring a robust adhesion of the coated material to the substrate. The nickel foil functioned as a dissemination/current gatherer for electrolytic ions. The selected substrate received a coating of 4.8 mg of the carbon material, resulting in a layer with dimensions of 1 × 1 cm^2^ and a thickness of 10 μm.

The EIS studies were carried out via a triple-electrode cell setup, using a 3M KOH solution serving as the electrolyte at ambient condition. This process was facilitated by a Biologic SP-200 Potentiostat/Galvanostat electrochemical analyser. The setup consisted of an activated carbon (AC) electrode material concealed on a selected nickel substrate serving as the functioning electrode, a saturated calomel electrode (SCE) chosen for the referral electrode, and after that, we chose a platinum wire for a counter electrode. Finally, the selected electrodes were submerged in 2 M KOH electrolyte solution, through a porous membrane barrier strategically placed between them to prevent short-circuiting. The characterization techniques employed an electrochemical impedance analyser (EIS) and cyclic voltammetry (CV) curves, and included galvanostatic charge discharge (GCD) tests using the specific capacitance which was calculated using the following formula (Equation (1)):

(1) $$Cs=\frac{I\times \Delta t}{m\times \Delta V}\;(F/g)$$

where Equation (1) C*s* is the specific capacitance (F/g), I is the discharge current(A), Δt is the discharging time(sec), (m) is the mass of active material (g), and ΔV is the potential window (V).

The specific energy density (ED), calculated in watt-hours per kilogram (Wh/kg), performance is determined using Equation (2).

(2) $$E_{D}=\frac{1}{2}\times {C_{s}(∆V)}^{2}\times \frac{1}{3.6}(\mathrm{Wh}/\mathrm{kg})$$

Similarly, the specific power density (PD), measured in watts per kilogram (W/kg), is another key performance indicator for ZISCs and is calculated using Equation (3).

(3) $$P_{D}=\frac{E_{D}\times 3600}{\Delta t}(W/\mathrm{kg})$$

where E_D_ is energy density (Wh/kg), P_D_ is power density (W/kg), Cs is specific capacitance of the device (F/g), ΔV is voltage window (V), Δt is discharge time (s).

## 3. Result and Discussion

### 3.1. XRD Analysis

**Figure 1:**
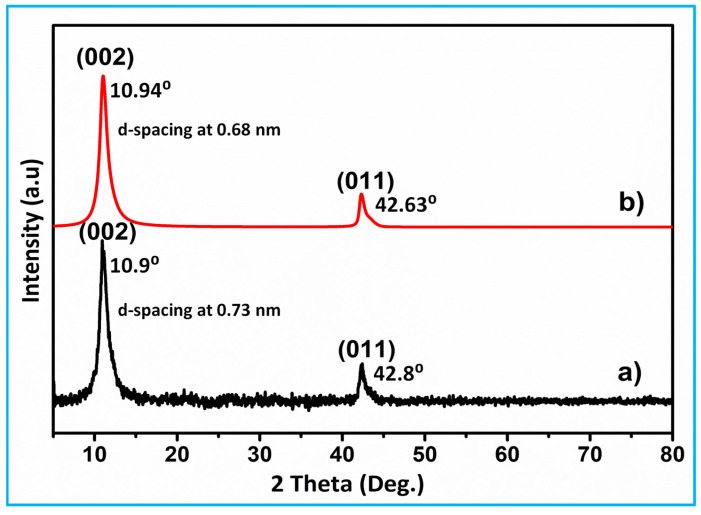
XRD graph of (**a**) SCB (AC) and (**b**) after activation (SCB-KOH-AC) materials.

The crystalline phase nature and size of the synthesized material were characterized via XRD studies. The XRD graph of prepared activated carbon (AC) and KOH-activated carbon samples are illustrated in Figure 1a. The XRD spectrum of SCB-AC powder exhibits a narrow peak at 2θ = 10.9° (d-spacing at 0.73 nm) and a small peak at 2θ = 42.8° which belong to the (002) and (011) hexagonal lattice planes of sp^2^ carbon [11]. This may be due to the intercalation process occurred by oxygen functional groups between the layers of carbon. This intercalation process increased the d-spacing value of activated carbon as 0.68 nm and implied the good oxidation of carbon to KOH-activated carbon. This implies the successful formation of activated carbon. The XRD spectrum (Figure 1b) shows a carbon peak corresponding to the KOH-activated carbon, which was previously not observed. The appearance of a new broad peak at 10.94° indicates that the activation process using KOH significantly altered the carbon structure, resulting in effective porosity and surface area enhancement through chemical activation rather than a complete reduction process. Furthermore, the XRD peak arose at around 2θ = 42.63° (011) in Figure 1b which may be due to crystalline disorder [12]. Additionally, the observed peaks are weak and broad, which demonstrates a crystalline form of the carbon network. In contrast, the SCB-KOH-AC sample, which underwent additional KOH chemical activation and purification, shows a slightly increased noise level in its XRD pattern, likely due to residual surface features and finer crystallite structures.

The Debye–Scherrer formula is a fundamental equation used in X-ray diffraction (XRD) to estimate the size of crystallites in a material. It is given by D = Kλ/βcosθ, where D-is the crystallite size, K is the shape factor, typically around 0.9. λ is the wavelength of the X-ray used in the diffraction, β is the full width at half maximum (FWHM) of the diffraction peak in radians, θ is the Bragg angle, which is the angle between the incident X-rays and the scattering planes. Figure 1a,b in the XRD patterns in the image show distinct diffraction peaks for two samples. Sample (a) exhibits peaks at 2θ = 10.9° and 42.8°, corresponding to the (002) and (011) planes, respectively, with a d-spacing of 0.73 nm. In comparison, sample (b) shows sharper peaks at 2θ = 10.94° and 42.63°, corresponding to the (002) and (011) planes, respectively, indicating a more ordered structure with a reduced d-spacing of 0.68 nm. The analyses revealed that the crystallite sizes of the activated carbon and the KOH-activated carbon were 18.3 nm and 14.7 nm, respectively. The reduction in crystallite size suggests that the activation process with KOH has refined the crystallite structure, making the grains smaller. This can be due to the etching effect of SCB with KOH, which removes amorphous carbon and creates a more defined crystalline structure for catalysis and energy storage applications.

### 3.2. FT-Raman Analysis

**Figure 2:**
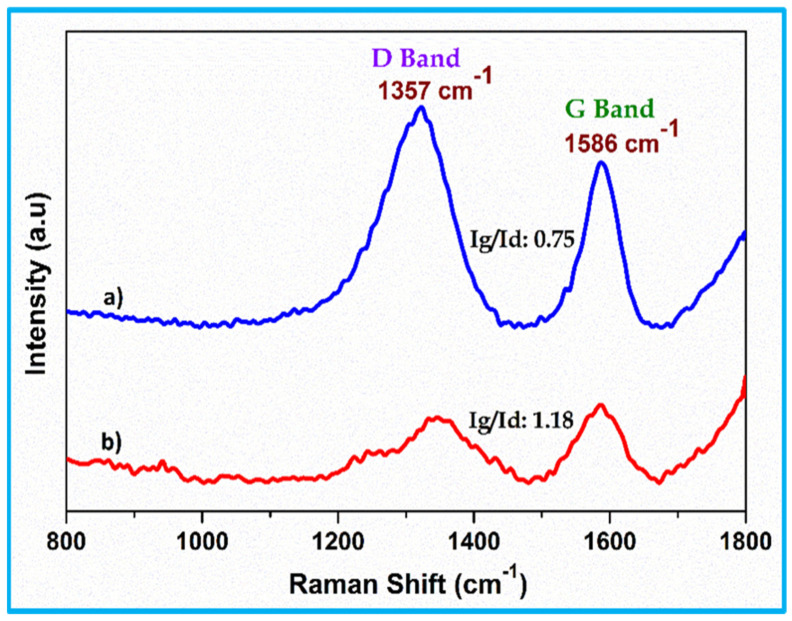
FT-Raman spectrum of (**a**) activated carbon (SBC-AC) and (**b**) KOH-activated carbon (SBC-KOH-AC) electrodes.

The Raman spectroscopy instrument is an important tool for analysing the structure, bonding, and order/disorder of carbon-based materials, including prepared SCB-AC and SCB-KOH-AC. Figure 2a,b depict the characteristic Raman spectra of these materials. The peak detected around 1357 cm^−1^ tends to the D band, which originates from the in-plane vibrational mode associated with defects and disordered sp^3^-hybridized carbon atoms. In contrast, the G band, positioned at approximately 1586 cm^−1^, stems from the in-plane stretching vibrations of sp_2_-hybridized carbon atoms in the graphitic structure of the activated carbon sheets [13]. This G band represents the second-order overtone of a different in-plane vibration. The presence of the D band signifies the existence of defects and disorder within the activated carbon structure. The ratio of the D band intensity (I_d_) to the G band intensity (I_g_), known as the I_g_/I_d_ ratio, can be employed as a semi-quantitative measure of this disorder. In this case, the I_g_/I_d_ value of 0.75 suggests a significant degree of lattice imperfections within the activated carbon sample (Figure 2a). The Raman spectrum of the KOH-activated carbon reveals several key differences compared to the original activated carbon (Figure 2b). Firstly, both the ‘D’ and ‘G’ band intensities exhibit variations, suggesting changes in the material’s defect concentration and graphitic ordering [14]. Additionally, there is a slight shift in the peak positions towards higher wavenumbers.

Secondly, the intensity ratio (ID/IG) for the KOH-activated carbon reaches 1.17. This significant increase compared to the original activated carbon indicates a successful activation process, potentially leading to a material with enhanced porosity and surface area. This finding aligns with the previous confirmation of structural changes achieved through XRD and FT-Raman analysis.

### 3.3. X-Ray Photoelectron Spectroscopy Analysis

**Figure 3:**
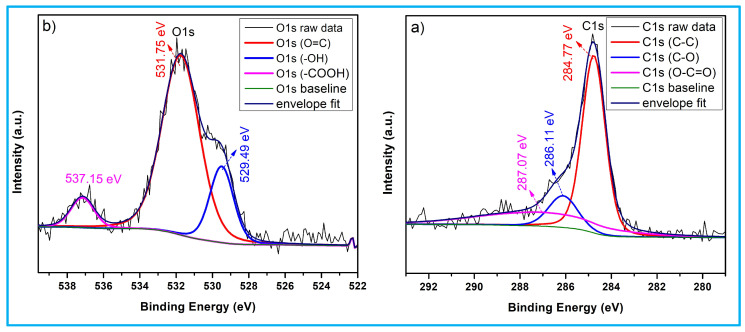
XPS spectra of activated carbon (SCB-KOH-AC) (**a**) O1s and (**b**) C1s.

Figure 3 illustrates the XPS analysis performed to investigate the electronic binding energy characteristics of SCB-AC and SCB-KOH-AC. In Figure 3a, the high-resolution C1s spectrum shows a dominant peak centered at 284.80 eV, corresponding to sp^2^-hybridized carbon (C–C). Using CASA-XPS software with Shirley background subtraction, the C 1s spectrum was deconvoluted into three main peaks at 284.77 eV, 286.11 eV, and 287.07 eV, attributed to C–C (sp^2^), C–O, and O–C=O functional groups, respectively. Figure 3b presents the O 1s spectrum, with peaks observed at 529.49 eV, 531.75 eV, and 537.15 eV, which are assigned to C=O (carbonyl), C–O (ether or hydroxyl), and –COOH/adsorbed H_2_O, respectively. These results indicate partial reduction of oxygen-containing functional groups during the activation process [15]. Furthermore, the enhanced intensity of the C=C peak suggests improved graphitization in the carbon framework of the synthesized materials.

### 3.4. Morphology Analysis

**Figure 4:**
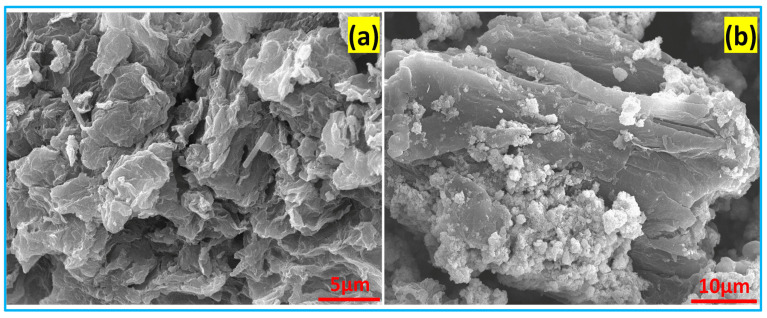
Morphology analysis of (**a**) activated carbon (SBC) and (**b**) KOH-activated carbon (SBC-KOH-AC).

The surface morphological analysis of the prepared AC and KOH-AC under augmented conditions and characterized SEM is presented in Figure 4a,b. Figure 4a illustrates that the micrograph of activated carbon shows a flake-like morphology. The particles appear to be layered or stacked in a slightly random orientation. The surface texture seems relatively smooth, with distinct, thin layers of material visible [16]. The particles are relatively small, with visible edges and boundaries between the individual flakes. Figure 4b demonstrates the KOH-activated carbon which shows a more complex, heterogeneous structure with a combination of larger flakes and smaller particulate matter. The surface appears rougher compared to Figure 4a, with the presence of both large flakes and smaller aggregated particles. In addition, in Figure 4a, these particles are larger compared to notable difference in size between the flakes and the smaller particulates adhered to them. This kind of slight difference in magnification, morphology, and texture suggests that these images could be showing different stages of a material’s synthesis or processing [17]. However, these observations are crucial in understanding the material’s properties, such as its mechanical strength, thermal conductivity, or potential applications in composites.

### 3.5. Elemental Analysis

**Figure 5:**
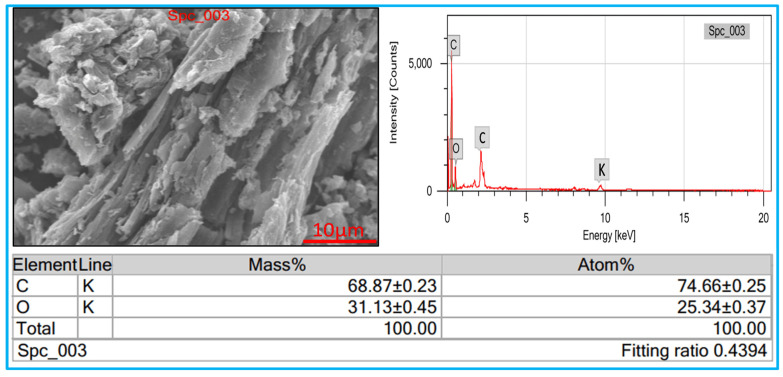
Elemental analysis of KOH-activated carbon (SCB-A).

In this investigation, Figure 5 shows the EDS spectrum of synthesized KOH-activated carbon; it shows that the oxygen (O) and carbon (C), additionally in the energy value from 2 to 3 keV carbon peak, appeared in the prepared materials and indicates the high purity of the prepared samples [18].

### 3.6. UV-DRS

**Figure 6:**
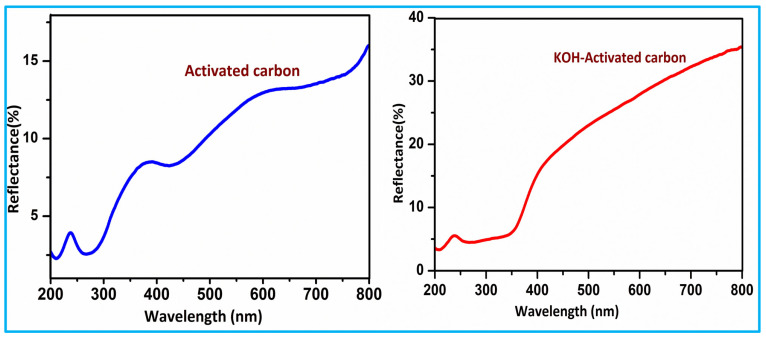
UV-DRS analysis of activated carbon and KOH-activated carbon.

**Figure 7:**
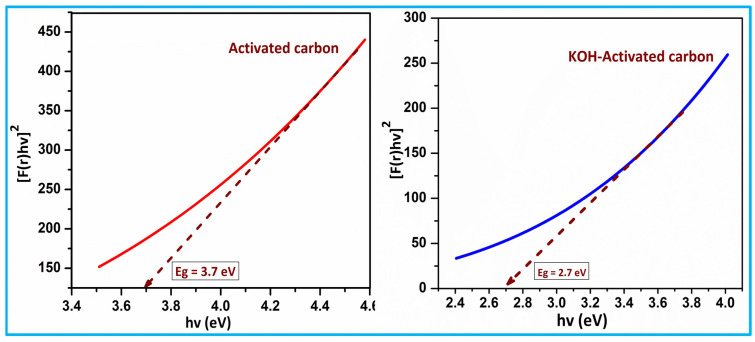
Tauc’s plot of activated carbon (AC) and KOH-activated carbon (KOH-AC).

UV–Visible diffuse reflectance spectroscopy of activated carbon and KOH-activated carbon materials. As shown in Figure 6, pure activated carbon nanoparticles had significant UV absorption edge observed at 800 nm, but the UV absorption of KOH-activated carbon samples shifted towards the higher wavelength side [19]. The changes in the absorption edges show the changes in the band structure. Further, as shown in Figure 7, the band gap of samples is determined by the Kubelka–Munk function equation (Equation (4)). The band gap of the prepared materials was found to be 3.7 and 2.7 eV, respectively [20].

(4) $$f(R)=\alpha /s={\left(1-R\right)}^{2}/2R$$

In this text, f(R) stands for the K-M function, α represents the absorption coefficient, s denotes the scattering coefficient, and R represents the reflection coefficient.

## 4. Electrochemical Measurements

### 4.1. Three-Electrode Cell System of CV, GCD, and EIS

**Figure 8:**
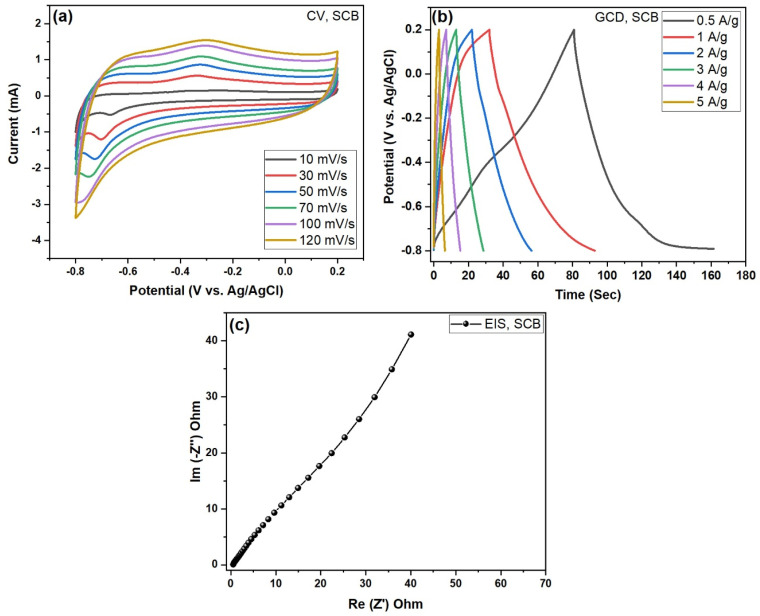
(**a**–**c**). Electrochemical performance of pure sugarcane bagasse (SCB)-derived activated carbon electrodes in a three-electrode configuration: (**a**) CV curves recorded at scan rates ranging from 10 to 120 mV/s, (**b**) GCD profiles at various current densities (0.5 to 5 A/g), and (**c**) electrochemical impedance (EIS)—Nyquist plot under potentiostatic conditions.

**Figure 9:**
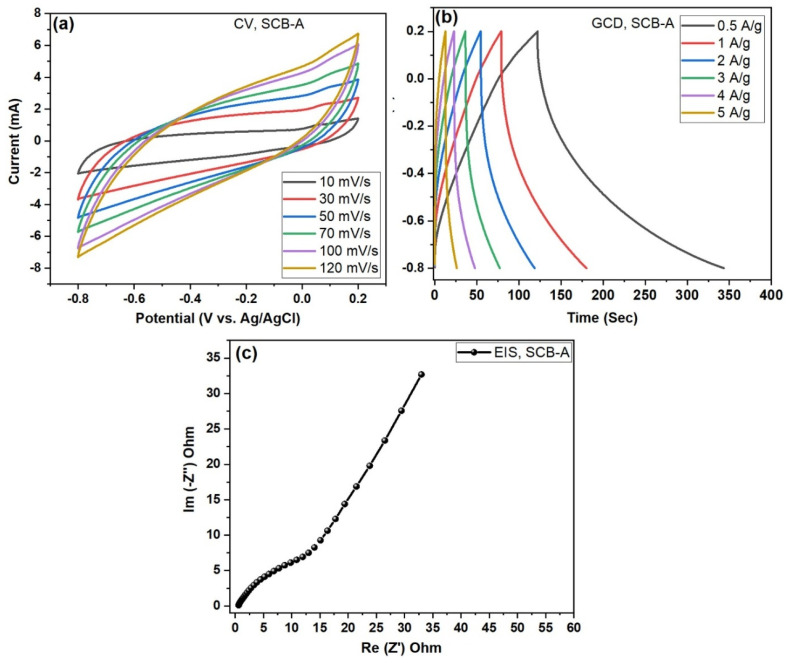
Electrochemical analysis of SCB-A electrode active material in a three-electrode system: (**a**) CV at different scan rates, (**b**) GCD at various current densities, (**c**) Nyquist plot from EIS measurements.

The electrochemical implementation of the prepared carbon electrodes is executed via CV, EIS, and GCD shown in the SCB carbon electrode in Figure 8a–c and in Figure 8 the SCB-A electrode. The electrochemical performance was carried with −0.2 to −0.8 V using the 3M KOH electrolyte solution. Figure 8a and Figure 9a illustrate both electrodes. Three electro-cells demonstrate the electrochemical analysis results of the activated carbon and KOH-activated carbon electrodes with different scan rate ranges from 10 to 120 mV/s [21].

From Figure 8a and Figure 9a, cyclic voltammetry (CV) of both the pure sugarcane bagasse (SCB) and KOH-derived activated carbon (SCB-A) electrode was recorded at scan rates of 10, 30, 50, 70, 100, and 120 mV/s. The CV curves maintained a nearly perfect rectangular shape across all scan rates. The shape of the voltammogram remained the same even at the higher scan rate, which indicates the rapid transfer of electrons towards the scan rate and shows the excellent reversibility of the electrode [22]. Figure 8a displays the cyclic voltammetry (CV) curves of the SCB electrode at various scan rates (10–120 mV/s) within a potential window of –0.8 to 0.2 V (vs. Ag/AgCl). The curves exhibit quasi-rectangular shapes with slight redox humps, indicating a major combination of electric double-layer capacitance (EDLC) and minor redox behavior. Due to the suggesting inadequate redox activity, the observed redox peaks are indirect, which may be attributed to the use of a Ni-foil conductive substrate combined with the pure carbon material. The near-symmetric shape of the CV profiles at all scan rates suggests good capacitive reversibility. These features are typical of carbon-based supercapacitor electrodes derived from biomass. From the CV curve, the typical oxidation and reduction peaks are observed in the SCB and SCB-A electrodes indicating the electric double-layer (E_DL_) nature of the electrodes in Figure 9a. The results suggest excellent capacitive behavior and fast ion transport within the maximum current response with respect to scan rate. This behavior confirms the ideal electric double-layer capacitance (EDLC) nature of the material with KOH-activated carbon electrode. This may be the interactive stimulate of the KOH in the KOH-activated carbon electrode [23].

The specific capacitance (F/g) values of the prepared carbon electrodes were calculated and scheduled in Equation (1). Activated carbon (SCB) and KOH-activated carbon (SCB-A) electrodes are displayed in GCD curves for the specific capacitance. Figure 8b and Figure 9b show the galvanostatic charge–discharge (GCD) profiles with the specific capacitance Csp (F/g) values of the prepared electrodes. The GCD curves were obtained at current densities ranging from 0.5 to 5 A/g. The triangular shape of the discharge curves reflects typical EDLC behavior with good reversibility. Figure 8b shows the GCD curves for pure SCB carbon electrodes; the specific capacitance values were calculated as 132.20, 93.26, 61.95, 38.34, 21.93, and 14.91 F/g at 0.5, 1, 2, 3, 4, and 5 A/g, respectively. Further, in Figure 9b, KOH-activated (SCB-A) carbon electrodes demonstrated the specific capacitance Csp—253.41, 173.88, 131.30, 85.11, 41.73, and 20.85 F/g, respectively. So, finally comparing (Table 1) both GCD curves, the maximum specific capacitance Csp = 132.20 F/g and 253.41 F/g corresponds with electrode active materials SCB and SCB-A, respectively. It can be clearly shown that the electrode is much higher than activated carbon specific capacitance (F/g) values, charging and discharging time for interaction between the electrode and electrolyte solution during the electrochemical performance [24]. A decrease in specific capacitance with increasing current density is attributed to limited ion diffusion at higher rates. These values demonstrate efficient charge storage performance at lower current densities [25]. However, it is much greater for the SCB-KOH-AC electrode than the pure electrode.

Potentiostatic electrochemical impedance spectroscopy (EIS) measurements were carried out for both pure sugarcane biomass-derived carbon (SCB) and KOH-activated SCB-A electrodes. The analysis was performed in the frequency range of 100 kHz to 1 kHz using a 10 mV with AC amplitude. As shown in Figure 8c, the Nyquist plot of the SCB electrode displays a semicircle in the high-frequency region, indicating solution resistance (Rs) and charge transfer resistance (Rct), and a linear region at lower frequencies with a ~45° slope, characteristic of Warburg impedance. The measured Rs and Rct values for SCB are 1.20 Ω and 19.76 Ω, respectively, suggesting moderate ion diffusion and interfacial resistance. In comparison, Figure 9c shows the Nyquist plot for SCB-A, which exhibits a steeper linear slope with lower resistance values along the x-axis and greater capacitance behavior along the y-axis. The SCB-A electrode shows improved electrochemical characteristics, with reduced Rs and Rct values of 0.60 Ω and 10.97 Ω, respectively, indicating enhanced charge transfer kinetics and ion accessibility.

Overall, biomass SCB-derived activated carbon is a sugarcane bagasse (SCB)-derived activated carbon that demonstrates promising performance as an electrode material in a three-electrode supercapacitor setup, as shown in Figure 8a–c and Figure 9a–c. The SCB electrode exhibits typical EDLC behavior with stable, rectangular CV curves and symmetric GCD profiles. KOH activation (SCB-A) significantly enhances electrochemical properties by increasing surface area and conductivity. SCB-A shows a higher current response across scan rates and improved capacitance performance. EIS results confirm lower charge transfer resistance (Rct: 10.97 Ω) compared to pure SCB (Rct: 19.76 Ω). This indicates more efficient ion diffusion and better electrode–electrolyte interaction. The improved performance is attributed to KOH-induced porosity development. Overall, SCB-A is a highly efficient electrode material for high-performance supercapacitor applications.

### 4.2. Electrochemical Performance of Two-Electrode Symmetric Device Configuration

The sugarcane bagasse (SCB)-derived activated pure carbon has gained significant attention due to its abundance, low cost, and high carbon content, making it a sustainable precursor for supercapacitor electrode materials. The activated carbon derived without chemical activation (SCB) exhibits a porous structure beneficial for charge storage. To enhance the electrochemical properties, chemical activation using potassium hydroxide (KOH) was employed to prepare SCB-A, resulting in improved surface area and conductivity. The electrochemical performance of these materials was evaluated in a two-electrode symmetric device configuration. The symmetric devices constructed using SCB//SCB and KOH-activated SCB-A//SCB-A electrodes were fabricated and tested. The SCB-A-based device demonstrated enhanced capacitive behavior compared to the pure SCB-based device. This improvement is primarily attributed to better ion accessibility and higher double-layer capacitance in SCB-A.

**Figure 10:**
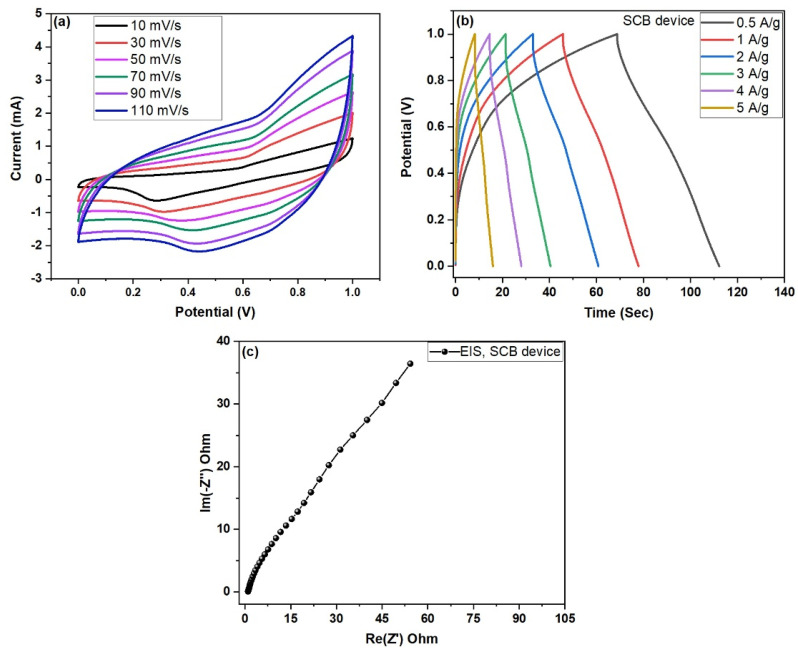
(**a**–**c**) Electrochemical performance of two-electrode symmetric device SCB//SCB pure AC; (**a**) CV curves at various scan rates, (**b**) GCD profiles at different current densities, and (**c**) wlectrochemical impedance spectroscopy (EIS) Nyquist plots for SCB devices.

**Figure 11:**
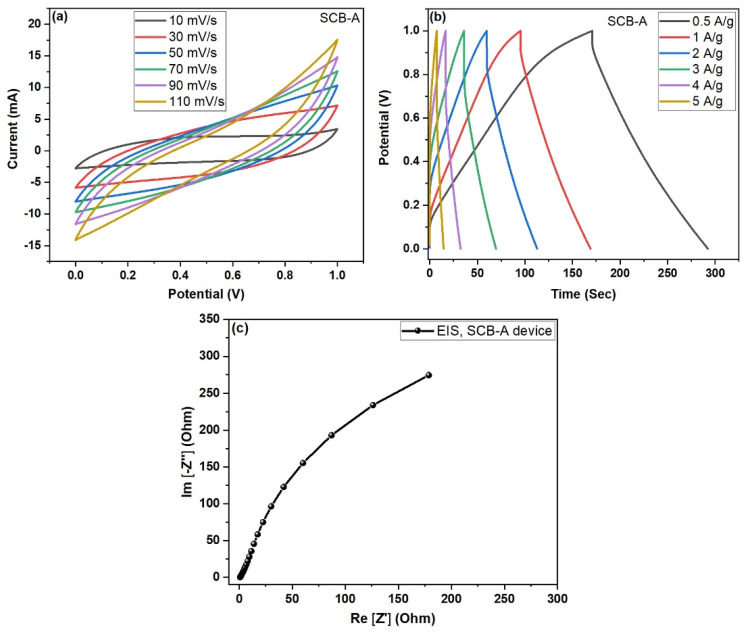
(**a**–**c**) Electrochemical performance of two-electrode symmetric device (KOH-activated carbon electrodes) SCB-A//SCB-A; (**a**) CV curves at different scan rates, (**b**) GCD curves at different current densities (A/g), and (**c**) electrochemical impedance spectroscopy (EIS) Nyquist plots for SCB-A devices, respectively.

In Figure 10a–c and Figure 11a–c, electrochemical measurements including cyclic voltammetry (CV), galvanostatic charge–discharge (GCD), and electrochemical impedance spectroscopy (EIS) were conducted to evaluate the device performance at fixed potential ranges from (0.0 to 1.0 V). CV curves indicated quasi-rectangular shapes, suggesting typical electric double-layer behavior. GCD profiles revealed good charge–discharge symmetry and stability at various current densities. EIS analysis showed lower equivalent series resistance (ESR) for the SCB-A//SCB-A device, confirming improved ion transport and conductivity due to KOH activation.

### 4.3. Electrochemical Performance of SCB//SCB Symmetric Device

Cyclic voltammetry (CV) was performed on the SCB//SCB symmetric supercapacitor within a potential window of 0–1.0 V using 3 M KOH aqueous electrolyte. As shown in Figure 10a, the CV curves exhibited a broad, nearly rectangular shape, characteristic of electric double-layer capacitance (EDLC), with a gradual increase in current response as scan rates increased. The charge–discharge (GCD) curves showed stable, symmetric triangular shapes, indicating excellent coulombic efficiency. Specific capacitance values calculated from GCD at current densities of 0.5, 1, 2, 3, 4, and 5 A/g were 84.64, 61.06, 47.65, 32.10, 19.73, and 9.81 F/g, respectively. As expected, capacitance decreased with increasing current density due to limited ion diffusion at higher rates. The internal resistance of the electrodes with the interaction of electrodes and electrolytes were characterized through EIS spectra. The electrochemical impedance spectroscopy (EIS) shown in Figure 10c presented a solution resistance (Rs) of 0.99 Ω and charge transfer resistance (Rct) of 17.28 Ω. The higher frequency region revealed Warburg impedance, confirming good ion diffusion [26,27]. Overall, the pure SCB//SCB device demonstrated stable electrochemical behavior with reliable capacitive properties.

### 4.4. Electrochemical Performance of Activated SCB-A//SCB-A Symmetric Device

The KOH-activated SCB-A//SCB-A symmetric device was also tested under identical conditions using 3 M KOH electrolyte. The CV curves shown in Figure 11a displayed typical EDLC-like behavior, without noticeable redox peaks, and showed an enhanced current response at higher scan rates, suggesting better electrochemical activity. The GCD curves in Figure 11b were symmetric and stable across all current densities, indicating highly reversible charge–discharge processes. From the Table 2, calculated specific capacitance values were significantly higher, measured as 145.21, 103.06, 81.44, 68.01, 51.80, and 37.91 F/g at 0.5, 1, 2, 3, 4, and 5 A/g, respectively. This improvement is attributed to the increased surface area and porosity resulting from KOH activation [28,29]. The EIS Nyquist plot in Figure 11c demonstrated a solution resistance (Rs) of 0.80 Ω and a charge transfer resistance (Rct) of 10.97 Ω, confirming enhanced ion transport and reduced internal resistance. These results highlight the superior capacitive and kinetic performance of the SCB-A//SCB-A device.

Finally, the SCB-A//SCB-A symmetric device outperformed the SCB//SCB device in all electrochemical tests. The maximum specific capacitance of SCB-A (145.21 F/g) was significantly higher than that of SCB (84.64 F/g), indicating the effectiveness of KOH activation. The enhancement is due to increased porosity, larger surface area, and more electrochemically active sites provided by the SCB-KOH-AC electrodes. CV and GCD analyses confirm improved charge storage ability and better reversibility in SCB-A. Additionally, EIS data show a lower Rct value of 10.97 Ω for SCB-A compared to 17.28 Ω for SCB, indicating faster charge transfer at the electrode–electrolyte interface. The reduced Rs and Warburg impedance in SCB-A further support superior ion diffusion. These factors collectively demonstrate that KOH-activated biomass carbon (SCB-A) is a more efficient and high-performing electrode material for symmetric supercapacitor applications. which aligns with typical activated similar carbon-based electrodes reported in the literature [30,31,32,33,34]. Table 2 demonstrates the electrochemical specific capacitance of SCB//SCB and SCB-A//SCB-A symmetric devices.

### 4.5. Cycling Stability and Capacity Retention (CR%)

The long-term cycling stability of both SCB//SCB and SCB-A//SCB-A symmetric devices was evaluated over 10,000 charge–discharge cycles. The SCB-A//SCB-A device exhibited excellent cycling performance with a capacity retention of over 98.43%, demonstrating superior durability. In contrast, the SCB//SCB device showed moderate stability, retaining around 93.89% of its initial capacitance. The enhanced stability of SCB-A is attributed to its improved structural integrity and conductive network from KOH activation. These results highlight the suitability of SCB-A as a promising electrode material for long-life energy storage applications. The GCD cycling performance confirms the superior electrochemical behavior of the KOH-activated carbon (SCB-A) electrode [35].

**Figure 12:**
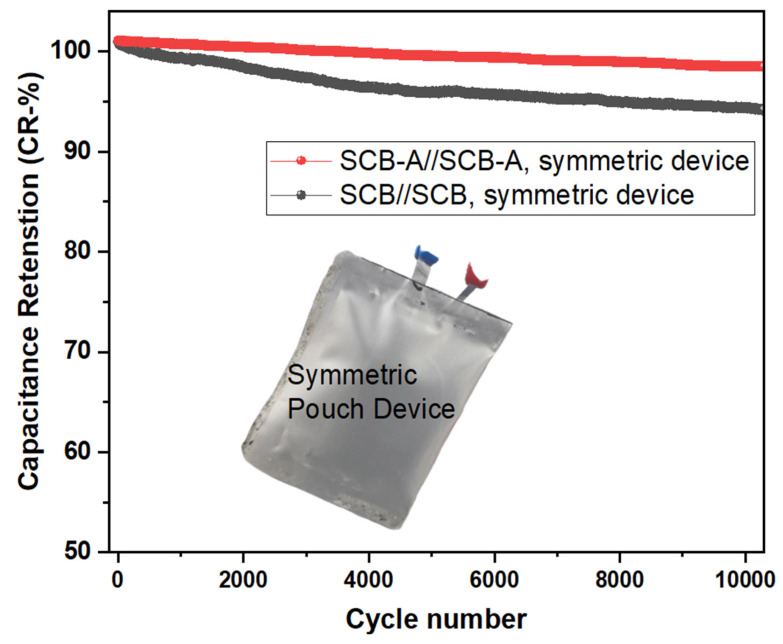
The electrochemical cycling stability and capacity retention (CR%) of SCB//SCB and SCB-A//SCB-A symmetric supercapacitor devices over 10,000 charge–discharge cycles at a constant current density 5 A/g.

Compared to pure SCB, the SCB-A electrode shows enhanced specific capacitance retention and long-term stability. This improvement is attributed to KOH activation, which increases conductivity and reduces internal resistance. Figure 12 illustrates the stable charge–discharge curves of both SCB and SCB-A electrodes during cycling tests. The SCB-A electrode also demonstrates better electrolyte wettability and chemical stability [36,37]. High capacitance retention (CR%) over extended cycles highlights its structural durability. The improved cycling stability results from the synergistic effects of KOH activation and pyrolysis [38]. These findings confirm SCB-A potential for high-performance energy storage in supercapacitor devices. Abundantly available sugarcane bagasse waste was utilized to produce pre-treated, activated carbon, demonstrating strong potential as a sustainable electrode material for aqueous-based supercapacitors and future large-scale industrial energy storage applications.

**Figure 13:**
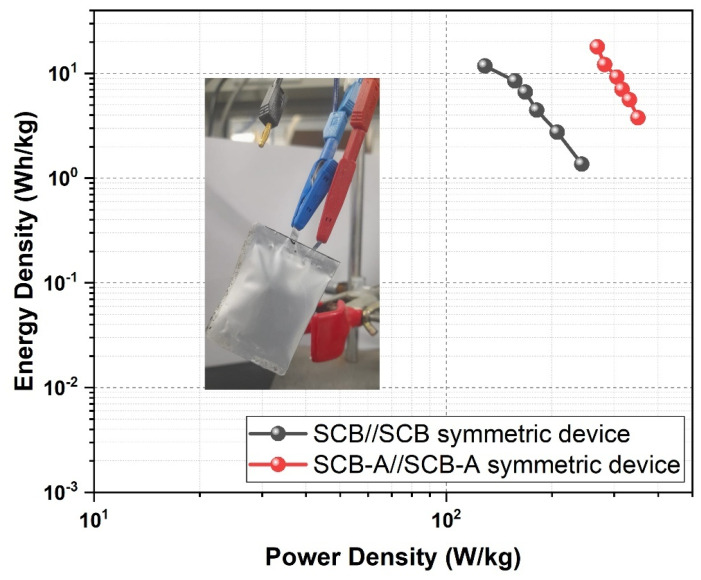
Energy and power density (Ragone plot) of SCB//SCB and SCB-A//SCB-A symmetric devices.

Figure 13, energy and power density corresponds to the E_D_, 75 Wh/kg and P_D_, 1290 W/kg, respectively. SCB-A//SCB-A symmetric devices show superior performance with energy density reaching. The SCB//SCB symmetric devices delivered the P_D_, 17.91 Wh/kg and power density up to P_D_, 2990 W/kg, using Equations (2) and (3). These results indicate a significant improvement in electrochemical performance upon activation of sugarcane bagasse. The high specific surface area and developed porosity in SCB-A materials contribute to enhanced ion accessibility. Compared to raw SCB carbon, SCB-A displays a better charge storage capability due to increased active sites. The activation with KOH effectively transforms sugarcane bagasse into a high-performance electrode material. In comparison, the symmetric SCB-A//SCB-A device demonstrates both higher energy and power densities than the SCB//SCB configuration. These findings highlight the potential of KOH-activated sugarcane bagasse-derived porous carbon for advanced supercapacitor applications.

## 5. Conclusions

In this work, a facile pyrolysis strategy was successfully employed to synthesize carbon materials from sugarcane bagasse (SCB), resulting in SCB-AC and KOH-activated SCB-AC (SCB-KOH-AC) electrodes for energy storage applications. The pure SCB-AC and KOH-activated SCB-AC materials were successfully synthesized, exhibiting crystallite sizes of 18.3 nm and 14.7 nm, respectively, with a layered or partially disordered stacking structure. These structural features demonstrate that the employed thermochemical approach is a simple and effective method for producing highly crystalline, bio-derived nanocarbons. The Kubelka–Munk function analysis revealed optical band gaps of 3.7 eV for SCB-AC and 2.7 eV for KOH-activated SCB-AC. Electrochemical testing in a three-electrode system showed specific capacitances of 132.20 F/g (SCB-AC) and 253.41 F/g (SCB-KOH-AC) at 0.5 A/g. Symmetric devices constructed with these materials delivered specific capacitances of 84.64 F/g (SCB//SCB) and 145.21 F/g (SCB-A//SCB-A). Notably, the SCB-KOH-AC device exhibited excellent cycling stability, retaining 98.43% of its capacitance after 10,000 cycles. These results clearly demonstrate the superior electrochemical performance of KOH-activated SCB-AC and underscore the potential of sugarcane bagasse as a sustainable precursor for high-performance supercapacitor electrodes.

## Figures and Tables

**Table 1:** Specific capacitance for activated carbon (SCB) and KOH-activated carbon (SCB-A) electrode using GCD curve (Figure 8b and Figure 9b).

Current Density A/g	Specific Capacitance (F/g)	
	(SCB) Activated Carbon	KOH-Activated Carbon (SCB-A)
0.5	132.20	253.41
1	93.26	173.88
2	61.95	131.30
3	38.34	85.11
4	21.93	41.73
5	14.91	20.85

**Table 2:** The specific capacitance calculated values for SCB//SCB and SCB-A//SCB-A symmetric devices electrodes for different current densities.

Current Density A/g	Specific Capacitance (F/g)	
	SCB//SCB Symmetric Device	SCB-A//SCB-A Symmetric Device
0.5	84.64	145.21
1	61.06	103.06
2	47.65	81.44
3	32.10	68.01
4	19.73	51.80
5	9.81	37.91

## Data Availability

The data presented in this study are available on request from the corresponding author.

## Supplementary Materials

The following supporting information can be downloaded at: https://www.mdpi.com/article/10.3390/nano15131028/s1, Figure S1: The TEM image represents 10 and 5 nm scale bar and it high resolution of visible lattice fringes (line dot markes inserted d-spcing and their planes); (a, b) SCB- AC and (C, d) SCB-KOH-AC; Figure S2: N_2_ adsorption–desorption isotherm with a narrow hysteresis loop (a) SCB-AC (b) SCB-KOH-AC indicating enhanced surface area and mesoporosity and BJH pore size versus pore volume distribution of (c) SCB-AC. (d) SCB-KOH-AC.
