# Supplementary material for: Sustainable Sugarcane Bagasse-Derived Activated Carbon for High-Performance Symmetric Supercapacitor Devices Applications
Source: Nanomaterials (Basel). 2025 Jul 2;15(13):1028. doi: 10.3390/nano15131028 (PMC12251455; doi:10.3390/nano15131028)
Supplement: Supplementary file 1 [file nanomaterials-15-01028-s001.zip › nanomaterials-3719256-supplementary.pdf]

Sustainable Sugarcane Bagasse-Derived Activated Carbon for High-Performance Symmetric Supercapacitor Devices Applications

Perumal Rajivgandhi [1,†], Vediyappan Thirumal [2,†], Alagan Sekar [1,*] and Jinho Kim [2,*]

[1] Department of Chemistry, Nehru Memorial College, Bharathidasan University, Puthanampatti, Trichy 621 007, Tamilnadu, India
[2] Department of Mechanical Engineering, Yeungnam University, Gyeongsan-si 38541, Gyeongbuk-do, Republic of Korea; thirumalvisnu@gmail.com
* Correspondence: alagansek66@gmail.com (A.S.); jinho@ynu.ac.kr (J.K.)
† These authors contributed equally to this work.

Supporting Information (SI)

Materials Characterization :

The characterization of the as-prepared SCB-AC and SCB-KOH-AC carbon materials was conducted to confirm their structural and surface morphological properties. The phase composition of the samples was analyzed using (Bruker-D2) PHASER powder X-ray diffractometer running at 40 kV with a current of 30 mA was used to evaluate powder X-ray diffraction (XRD) profiles were recorded in the 2θ range of 10° to 80°. FT-Raman spectroscopy was performed using an the spectra were acquired using a BRUKER ALPHA FT-Raman Spectrophotometer through opus 7.2 version software in the range from 800 to 1800 cm^{-1} . The Scanning Electron Microscope (SEM), surface morphologies of SCB-AC and SCB-KOH-AC were examined using a through JEOL JSM-6010LA scanning electron micrographs and coupled with a Bruker Energy Dispersive X-ray Spectroscopy (EDS) system (Czech Republic), operated at an accelerating voltage of 15 kV. UV–Vis spectroscopy was performed using a PerkinElmer UV–Vis spectrometer equipped with a wavelength range of 200–800 nm. This instrument enables accurate optical absorbance measurements for evaluating the electronic transitions and optical properties of the synthesized carbon materials. Further nanoscale

structural analysis was conducted using field emission transmission electron microscopy (FE-TEM) with an "FEI-TECNAI TF-20" at an accelerating voltage of 200 kV (supporting information section; Fig. S1). X-ray Photoelectron Spectroscopy (XPS) analysis was carried out using a ULVAC-PHI XPS VersaProbe III system. This instrument is equipped with a monochromatic Al Kα X-ray source (1486.6 eV) and operates under ultra-high vacuum conditions (~10^{-9} Torr). It provides high-resolution elemental and chemical state analysis with a spatial resolution down to 10 μm, ideal for surface characterization of carbon materials. The specific surface area, pore volume, and pore size distribution of the samples were determined using the Quantachrome NOVA 2200E BET Surface Area Analyzer (Autosorb-1-C-8, USA), which is a fully automated system capable of measuring BET surface areas as low as 0.1 m^2 (supporting information section; Fig. S2).

FE-TEM Suraface Characterization:

Fig. S1 presents high-resolution Field Emission Transmission Electron Microscopy (FE-TEM) images of SCB-derived carbon materials at 10 nm and 5 nm scales. The visible lattice fringes confirm the partial graphitic nature of the synthesized materials. In Fig. S1(a, b), the SCB-AC sample shows a well-defined lattice structure with a measured interlayer spacing of approximately 0.75 nm, attributed to the (002) plane of turbostratic carbon. The corresponding line-dot analysis in Fig. S1(b) confirms this spacing. Meanwhile, Fig. S1(c, d) displays the FE-TEM image of SCB-KOH-AC, where enhanced graphitic ordering is observed. The d-spacing measured from Fig. S1(d) is approximately 0.64 nm, also indexed to the (002) plane. This reduction in d-spacing after KOH activation suggests improved graphitization and structural ordering. These observations support the structural integrity and high surface reactivity of both carbon materials for energy storage applications.

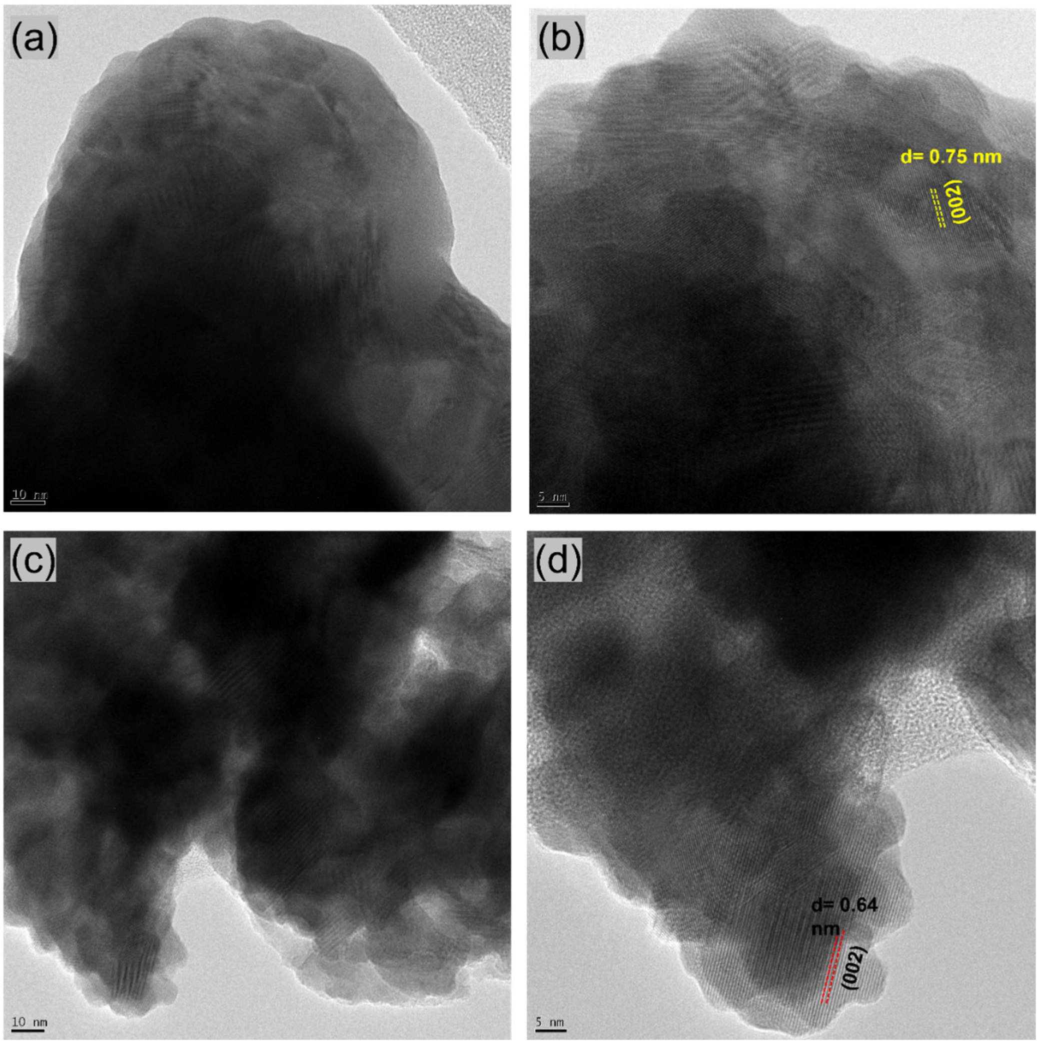

Fig. S1 The TEM image represents 10 and 5 nm scale bar and it high resolution of visible lattice fringes (line dot markes inserted d-spcing and their planes); (a, b) SCB- AC and (C, d) SCB-KOH-AC.

BET Surface Area and Pore Structure Analysis :

The N_2 adsorption–desorption isotherms of SCB-AC and SCB-KOH-AC are presented in Fig. S2(a) and (b), respectively. Fig. S2(a) SCB-AC (Fig. a) adsorption quantity at $P/P_0 \approx 0.3$: ~6.0 cm^3/g, SCB-AC exhibits a typical type IV isotherm indicates a lower BET surface area of 180 m^2/g with moderate adsorption capacity, indicating the presence of mesopores. Fig. S2 (b) SCB-KOH-AC (Fig. S2 (b)) adsorption quantity at $P/P_0 \approx 0.3$: ~3.0 cm^3/g (the sharp increase at high P/P_0), Due to high mesoporous structure and large hysteresis loop, indicating

micropores and mesopores, significantly higher BET surface area is 250 m²/g. So finally the BET surface area of SCB-AC is relatively low, consistent with its limited porosity. In contrast, SCB-KOH-AC shows a pronounced hysteresis loop and higher nitrogen uptake at high relative pressure, suggesting enhanced meso/microporous structure and significantly increased surface area. The pore volume distribution curves (Fig. S2 (c and d) further confirm this enhancement. SCB-AC shows a narrow pore distribution with a average mesopores peak pore size appears to be around ~45-50 nm. However, SCB-KOH-AC displays a broader and more prominent distribution, peaking at a strong peak appears around 22–25 nm, indicating superior pore development due to KOH activation. This structural improvement directly supports the enhanced electrochemical performance of SCB-KOH-AC.

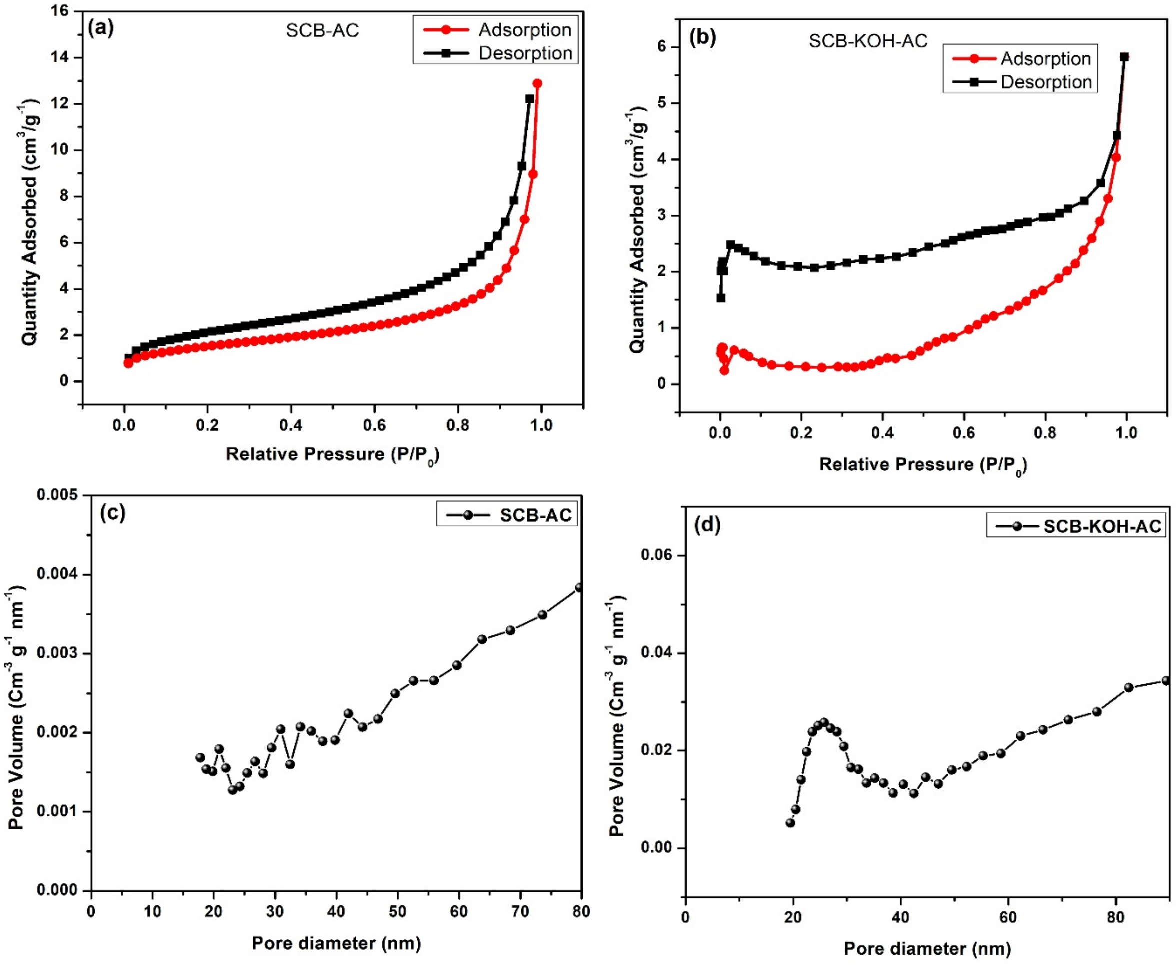

Fig. S2: N_2 adsorption–desorption isotherm with a narrow hysteresis loop (a) SCB-AC (b) SCB-KOH-AC indicating enhanced surface area and mesoporosity and BJH pore size versus pore volume distribution of (c) SCB-AC.(d) SCB-KOH-AC.
